# Weight Perturbation Alters Leptin Signal Transduction in a Region-Specific Manner throughout the Brain

**DOI:** 10.1371/journal.pone.0168226

**Published:** 2017-01-20

**Authors:** Michael V. Morabito, Yann Ravussin, Bridget R. Mueller, Alicja A. Skowronski, Kazuhisa Watanabe, Kylie S. Foo, Samuel X. Lee, Anders Lehmann, Stephan Hjorth, Lori M. Zeltser, Charles A. LeDuc, Rudolph L. Leibel

**Affiliations:** 1 Department of Pediatrics, Division of Molecular Genetics, Columbia University, College of Physicians and Surgeons, New York, New York, United States of America; 2 AstraZeneca, R&D Disease Area Diabetes/Obesity, Mölndal, Sweden; University of California San Francisco, UNITED STATES

## Abstract

Diet-induced obesity (DIO) resulting from consumption of a high fat diet (HFD) attenuates normal neuronal responses to leptin and may contribute to the metabolic defense of an acquired higher body weight in humans; the molecular bases for the persistence of this defense are unknown. We measured the responses of 23 brain regions to exogenous leptin in 4 different groups of weight- and/or diet-perturbed mice. Responses to leptin were assessed by quantifying pSTAT3 levels in brain nuclei 30 minutes following 3 mg/kg intraperitoneal leptin. HFD attenuated leptin sensing throughout the brain, but weight loss did not restore central leptin signaling to control levels in several brain regions important in energy homeostasis, including the arcuate and dorsomedial hypothalamic nuclei. Effects of diet on leptin signaling varied by brain region, with results dependent on the method of weight loss (restriction of calories of HFD, ad lib intake of standard mouse chow). High fat diet attenuates leptin signaling throughout the brain, but some brain regions maintain their ability to sense leptin. Weight loss restores leptin sensing to some degree in most (but not all) brain regions, while other brain regions display hypersensitivity to leptin following weight loss. Normal leptin sensing was restored in several brain regions, with the pattern of restoration dependent on the method of weight loss.

## Introduction

Many co-morbidities associated with obesity are mitigated by long-term maintenance of even modest (10%) body weight reduction [1]. However, the majority of formerly-obese individuals regain most or all of their lost weight [2], suggesting that powerful physiological (as well as environmental) mechanisms oppose the long-term maintenance of reduced body mass. Achievement and maintenance of reduced body weight in both mice [3] and humans [4] is accompanied by a significant decrease in energy expenditure that is ~15–20% greater than can be accounted for by changes in body mass and composition [3, 4]. This decrease in energy expenditure is due, at least in part, to the metabolic consequences of reductions in circulating leptin concentrations that are, in turn, the consequence of (and proportionate to) decreases in body fat.

Circulating leptin is the major afferent signal of somatic energy stores, with the hypometabolic phenotype of weight-reduced individuals resulting from, at least in part, a state of “perceived” relative leptin insufficiency [4, 5]. Reduced leptin signaling in the central nervous system causes decreased levels of circulating thyroid hormones, reduced sympathetic nervous system tone, increased skeletal muscle work efficiency (reflecting the consequences of changes in thyroid hormones and sympathetic nervous tone), and an increased drive to eat [3, 4, 6–9]. The administration of “replacement” doses of leptin to weight reduced humans [10–12] and rodents [13–15] rectifies most of these bioenergetic and behavioral phenotypes, confirming that brain sensing of leptin plays a critical role in mediating these responses to reduced body weight.

In mice, diet-induced obesity (DIO) results in either attenuation or elimination of cellular responses normally observed in leptin-sensitive circuits in the brain at normal weight [16, 17]. Continued failure of these circuits to respond appropriately to ambient leptin following weight loss suggests that these DIO-mediated modifications of the brain can result in cellular resistance to the actions of leptin which persist indefinitely in the weight-reduced brain [3].

In previous studies we described a mouse model of weight perturbation that mimics physiological and metabolic alterations to weight gain and loss observed in humans. Specifically, when they are calorically restricted, DIO and never-obese mice both show reductions in energy expenditure (adjusted for body mass and composition) [3, 18–20]. These responses are comparable (minus 10–15%) to those seen in weight-reduced human subjects; [4, 6]. The impact of weight perturbation on leptin signaling throughout the entire brain has not been analyzed with a high degree of accuracy. We hypothesized that these weight and diet perturbations would not only alter leptin signaling in previously identified feeding circuits, but that analysis of leptin signaling in an unbiased, comprehensive, manner would identify novel brain regions affected by these manipulations. We measured the ability of exogenous leptin to activate the long-form of the leptin receptor in 23 distinct brain regions in 4 different groups of mice: mice fed a control diet (10% calories from fat) *ad libitum* (LF); mice fed HFD (60% calories from fat) *ad libitum* (HF); mice fed HFD *ad libitum* followed by a period of caloric restriction (CR); and mice fed HFD and then switched back to control diet (LF-HF).

## Materials and Methods

### Animals

Beginning at 6 weeks of age, cohorts of male C57BL/6J mice were *ad libitum* fed either a high-fat diet (HFD) (60% kcal/fat, #D12492i, Research Diets, New Brunswick, NJ; N = 61) or a control (CON) diet (10% kcal/fat, #D12450Bi, Research Diets, New Brunswick, NJ; N = 18) for a period of 30 weeks, at which time they were divided into 4 groups for 4 additional months: **LF**–maintained on control diet ad lib (N = 18); **HF**–maintained on HFD ad lib (N = 16); **CR**–calorically restricted on HFD to maintain 20% weight loss (N = 20); **HF-LF**–switched from HFD to *ad libitum* control diet (N = 25). All mice were provided water *ad libitum* and maintained on a 12 hr light-dark cycle (lights on at 7 a.m.) for the duration of the experiment. Mice were injected with either leptin (3 mg/kg) or saline 30 minutes prior to perfusion with saline followed by 4% paraformaldehyde. This study was carried out in strict accordance with the recommendations in the Guide for the Care and Use of Laboratory Animals of the National Institutes of Health. All experiments were approved by the Columbia University Institutional Animal Care and Utilization Committee (AAALAC Accreditation #000687).

### Body Weight and Composition

Body weight was measured weekly using an Ohaus Scout Pro 200g scale with precision to a single decimal place (Nänikon Switzerland). Body composition (fat mass: FM; fat-free mass: FFM, & extracellular fluid) were measured on a mouse carcass-calibrated [21] time-domain-NMR (Minispec Analyst AD; Bruker Optics, Silberstreifen, Germany).

### Serum Glucose, Leptin and Insulin Concentrations

Blood was obtained by capillary tail bleed following a 4 hour fast (at 1:30PM) and glucose was measured with a glucometer (FreeStyle Lite, Abbott, Alameda, CA). Blood for hormone analysis was obtained by retro-orbital bleed following a 4-h fast, allowed to clot for 2 hours at room temperature, spun at 4°C for 20 min at 1000 × g, and serum was collected and frozen at −80°C. Leptin was assayed using the Quantikine ELISA kit (R&D Systems, Minneapolis, MN) according to the manufacturer’s instructions. Insulin was assayed using the Mercodia Ultrasensitive Mouse Insulin ELISA (Mercodia, Uppsala, Sweden); HOMA-IR was used to estimate insulin resistance and insulin sensitivity [22].

### Energy Intake and Expenditure

Food intake was measured by weighing food hoppers in group-housed cages (N = 3 cages per diet, 4–5 mice per cage) for LF, HF, and HF-LF mouse groups; the average daily food intake determined over a span of 4 days and calculated by multiplying the weight (in grams) by the caloric content of the diet (3.85 or 5.24 kcal/g for LFD and HFD, respectively). CR mice were initially fed 50% of their average *ad libitum* daily food intake until their body weight reached 80% of initial value at which time food was titrated to maintain 80% of initial body weight. All feeding was done twice daily with 1/3 of the food at 0900 and 2/3 at 1800 similar to previous studies by our group [3, 20]. Energy expenditure and movement was measured individually with a LabMaster-CaloSys-Calorimetry System (TSE Systems, Bad Homburg, Germany). O_2_ and CO_2_ measurements were taken every 26 min during a 72-h period while mice were provided their prescribed diet and *ad libitum* access water. Because of possible stress related to transfer to the chambers, only the last 48 h (of a total 72 h) of measurements were used to calculate total 24-h energy expenditure (TEE; expressed in kcal/day) and respiratory quotient (RQ = VCO2/VO2). Resting energy expenditure (REE in kcal/day) was defined as the lowest one hour period of energy expenditure, which coincided with the lowest one hour of total ambulatory activity during the 48-h period; this value was extrapolated to 24 h. Physical activity was measured by an infrared beam system integrated with the LabMaster System. Total activity (beam breaks) in x-, y-, and z-axis was stored every 14 min. The system is designed to differentiate between fine motor movement (defined as a single x- or y-axis beam break), ambulatory movement (defined as the simultaneous breaking of two adjacent x- or y-beams), and rearing, defined as the breaking of the z-axis infrared beam.

Non-resting energy expenditure (NREE) was calculated as the arithmetic difference between TEE and REE. Regression analysis relating energy expenditure parameters (i.e. TEE, REE, NREE) to FM and FFM (combined) were calculated using HF and LF mice. These equations were used to predict EE parameters for all mice following experimental weight perturbation as we have done in similar studies [3]. Residuals were calculated as the difference between measured and predicted values for each mouse.

### Immunohistochemistry

Mice were fasted overnight (~ 16 hours) and injected intraperitoneally with either 3 mg/kg leptin or 0.9% saline 30 minutes prior to perfusion with 4% PFA; brains were removed and cryoprotected in 30% sucrose prior to embedding in OCT Compound (Sakura Finetek USA, Torrance, CA). Coronal sections (ranging from +2.0mm through -8.0mm Bregma) were obtained in eight series of 20μm sections. Free-floating sections from a single series were incubated overnight at 4°C with primary antibody targeting phosphorylated STAT3 (1:2,500; #9131, Cell Signaling Technology, Danvers, MA). Sections were then washed, incubated with biotinylated goat anti-rabbit secondary antibody (1:500; Vector Laboratories, Burlingame, CA) for 1 hour, and processed for colorimetric staining with VECTASTAIN elite ABC reagent and DAB Peroxidase Substrate (Vector Laboratories, Burlingame, CA) according to manufacturer’s instructions. For each animal, IHC was performing on free-floating sections, so all section for a given animal were stained simultaneously. For all animals, IHC was performed in batches of 12, with at least one member of each experimental group present in each batch; both leptin- and saline-treated LF samples were present in all batches as positive and negative controls. There were no batches in which staining for all animals was notably higher/lower than other batches. Sections were counterstained with hematoxylin QS reagent (Vector Laboratories, Burlingame, CA) to aid mapping of brain regions in relation to the neighboring neuroarchitecture. Following staining, sections were mounted on gelatin-subbed slides (Southern Biotechnology Associates, Birmingham, AL) and scanned at 40X using a high-throughput slide scanner (SCN400F; Leica Microsystems, Buffalo Grove, IL).

### Data Analysis

Images of stained brain sections were analyzed using HALO software (Indica Labs) to measure cell-by-cell pSTAT3 staining. Specifically, each brain region was identified according to the Allen Mouse Brain Atlas [23] and annotated for each section on a slide. Brain regions selected for analysis included the Nucleus Accumbens (ACB); Amygdala (Amg); Arcuate Hypothalamic Nucleus (ARH); Bed Nucleus of the Stria Terminalis (BST); Cochlear Nuclei (CO); Dorsomedial Nucleus of the Hypothalamus (DMH); Dorsal Raphe Nucleus (DR); Habenula (Hbn); Locus Ceruleus (LC); Medial Mammillary Nucleus (MM); Nucleus of the Solitary Tract (NTS); Periaqueductal Gray (PAG); Parabrachial Nucleus (PB); Premammillary Nucleus (PM); Principal Sensory Nucleus of the Trigeminal (PSV); Paraventricular Nucleus of the Hypothalamus (PVH);Paraventricular Nucleus of the Thalamus (PVT); Suprachiasmatic Nucleus (SCH); Subfornical Organ (SFO); Substantia Nigra pars compacta (SNc); Subthalamic Nucleus (STN); Supramammillary Nucleus (SUM); and Ventromedial Hypothalamic Nucleus (VMH) (S1 Table). As the HALO software allows for the individual analysis of multiple colorimetric stains based on the color of individual pixels in the image, each cell in a given brain region was individually assessed for nuclear positivity, classified as either negative (hematoxylin only) or positive for pSTAT3 (hematoxylin plus DAB) based on a manually input minimum optical density (OD) for the brown DAB stain (determined individually for each region of interest = ROI), and quantified for both cell nucleus size and staining intensity, with staining intensity normalized by subtracting sub-threshold DAB OD. Independent testing of unrelated brain sections confirmed that the addition of the hematoxylin counterstain did not affect the ability of the software to accurately identify DAB staining. The intensity of the DAB signal in a given cell, total intensity of a brain region, and intensity density of a brain region were determined by the following equations, where OD_Min_ represents the minimum OD threshold value (on a scale of 0–1) required for a cell nucleus to be considered positive for pSTAT3 staining:

IntensityCell = (ODCell – ODMin) / (1 – ODMin)

IntensityTotal = Sum of all IntensityCell in a Region

Density = IntensityTotal / Surface Area of Region

The mean pSTAT3 density for saline treated animals was subtracted from that of leptin-treated for each treatment group to determine the leptin-induced pSTAT3 signal for each group in each region. Due to large variations in scale for some brain regions versus most others (ie. the SFO and ARH display a much larger degree of activation than other brain regions), the data are presented to scale for each brain region individually to accurately convey the relative levels of activation within each brain region.

### Statistical Methods

Mouse body weight data was analyzed by two-way analysis of variance (ANOVA) with Bonferroni’s post-test. Glucose data were analyzed by the Kruskal-Wallis test with Dunn’s post-test. All other data were analyzed by one-way ANOVA with Tukey’s post-test. Only P values less than 0.05 were considered significant.

## Results

### Body Weight and Composition Following Diet and Weight Perturbation

**Fig 1:**
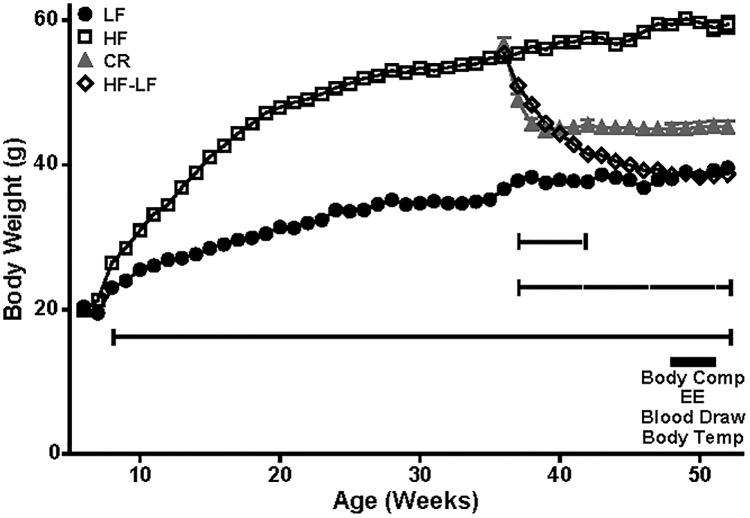
Body Weight and Experimental Design of Weight Perturbation Studies. Weight curves are presented for LF (solid circles; N = 18), HF (white squares; N = 16), CR (grey solid triangles; N = 20), and HF-LF (white diamonds; N = 25) mice (mean +/- SEM). Timing of physiological analyses is indicated by a black rectangle (48–51 weeks). Significant difference from LF is indicated by barred solid (HF), dashed (CR), and dotted lines (HF-LF); body weight of both CR and HF-LF groups were significantly different (P<0.0001) from HF mice at all time-points beside the initial time point for each mouse group. *** P<0.001.

**Fig 2:**
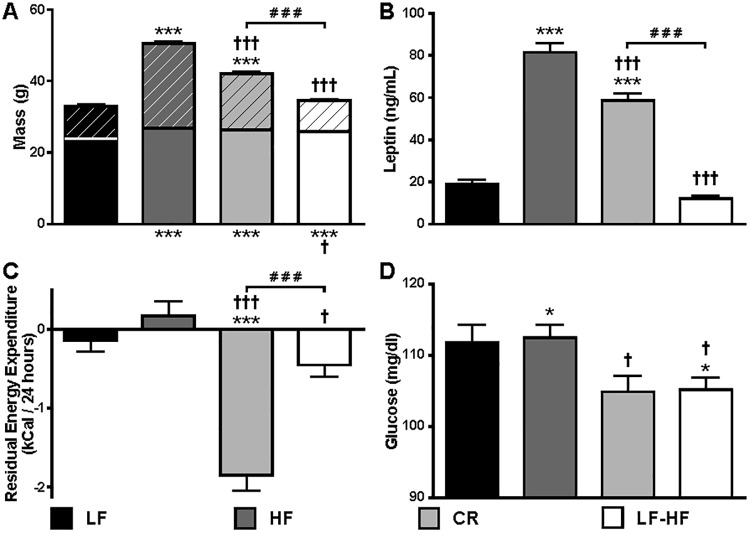
Body Composition, Circulating Leptin, Energy Expenditure and Fasting Glucose Concentrations of Weight Perturbed Mice. Mouse groups are indicated by black (LF, N = 18), dark gray (HF, N = 16), light gray (CR, N = 20), and white (HF-LF, N = 25) bars for all panels. Data provided are Mean +/- SEM of phenotypes as indicated in Fig 1. (A) Body composition analysis is presented as both fat-free mass (solid bars) and fat mass (diagonal bars); statistical significance of differences between mouse groups is indicated for FFM at the bottom and FM at top of each bar. (B) Serum leptin, (C) Residual energy expenditure (See Experimental Procedures), and (D) Fasting glucose concentrations. *** P<0.001 compared to LF; † P<0.05, ††† P<0.001 compared to HF; ### P<0.001 between weight reduced groups.

After HFD was introduced at 6 weeks of age, HF mice gained significantly more weight than LF mice (34.96 grams vs. 16.32 grams) over 30 weeks of *ad libitum* access to diet prior to initiation of weight perturbation (Fig 1). The increased body mass of HF mice resulted primarily from an increase in fat mass (83%), though fat-free mass was also significantly increased compared to LF mice (Fig 2A). After 12 weeks of *ad libitum* access to low-fat diet, the body weights of previously obese HF-LF mice were indistinguishable from those of never-obese LF controls (Fig 1); the majority of the weight loss of HF-LF mice was due to a reduction in fat mass (Fig 2A). CR mice, by design, were maintained at a body mass 20% below their starting mass (Fig 1), resulting in a fat mass intermediate between that observed for HF and LF mice (Fig 2A). Though both CR and HF-LF mice exhibited significant reductions in fat mass compared to HF mice, fat-free mass for each group remained significantly higher than LF mice (Fig 2A). Circulating leptin concentrations were directly proportional to fat mass (Fig 2A and 2B), indicating that all groups animals were in energy balance when assessments were performed [24].

### Energy Intake and Expenditure

Food intake and energy expenditure of weight- and diet-perturbed mice were compared to mice that had been continuously fed *ad libitum* (AL). Food intake for each mouse group was proportional to body weight (Fig 1) and composition (Fig 2A) (LF, 11.1±0.1 kcal/day; HF, 15.4±0.8 kcal/day (P<0.01 vs LF and HF-LF); CR, 12.7±0.3 kcal/day (P<0.05 vs LF); HF-LF, 10.8±0.3 kcal/day). A regression analysis relating FM and FFM to TEE was conducted using the AL mice (i.e. HF and LF groups) and residuals were calculated for all mice as previously described [3]. Adjusted for body mass and composition, CR mice were hypometabolic (negative residuals) compared to HF mice (P<0.0001; Fig 2C). HF-LF mice trended toward hypometabolism (P<0.14) compared to LF mice (Fig 2C). Locomotor activity was similar in all groups (data not shown).

### 4-hour Fasting Serum Glucose and Insulin; HOMA IR

Approximately 14 weeks after diet switch and/or weight reduction (Fig 1), animals that were weight stable for at least two weeks were fasted for 4 hours and capillary blood glucose was measured at 1:30PM. CR and HF-LF animals had lower fasting glucose concentrations (-6.78% and -6.47%, respectively) compared to HF mice (Fig 2D). Fasting glucose concentrations of HF-LF mice were lower than LF mice, and glucose concentrations of CR trended lower than LF levels (P<0.061; Fig 2D). HF mice did not have significantly higher fasting glucose concentrations than LF mice, but HF mice were hyperinsulinemic compared to LF and were more insulin resistant as assessed by HOMA2 IR. Return to control diet resolved these differences in HF-LF mice, but caloric restriction did not do so for CR mice (Table 1).

**Table 1:** Summary of Physiological Data.

	LF (n = 18)	HF (n = 16)	CR (n = 20)	HF-LF (n = 25)
Body Weight (g)	39.6 +/- 0.6	59.4 +/- 1.2***	45.2 +/- 0.8***	38.7 +/- 0.7
Fat Mass (g)	9.1 +/- 0.4	23.7 +/- 0.6***	15.9 +/- 0.5***	8.6 +/- 0.5
Energy Expenditure (kcal/day)	-0.13 +/- 0.14	0.17 +/- 0.19	-1.85 +/- 0.20*	-0.45 +/- 0.15*
Glucose (mg/dL)	111.9 +/- 2.5	112.5 +/- 1.8	104.9 +/- 2.3	105.2 +/- 1.7*
Plasma Leptin (ng/mL)	18.9 +/- 2.1	81.4 +/- 4.6***	58.6 +/- 3.5***	12.1 +/- 1.6
Plasma Insulin (ng/mL)	0.147 +/- 0.009	0.264 +/- 0.019*	0.320 +/- 0.041***	0.160 +/- 0.013
HOMA2 IR	0.51 +/- 0.03	0.91 +/- 0.07***	1.13 +/- 0.15***	0.54 +/- 0.04

Data are presented as mean ± SEM;

* P<0.05,

** P<0.01,

*** P<0.001 compared to LF, and were obtained the day before animals were sacrificed (with the exception of energy expenditure data, which were measured 3 weeks prior to sacrifice).

### Leptin Signaling

**Fig 3:**
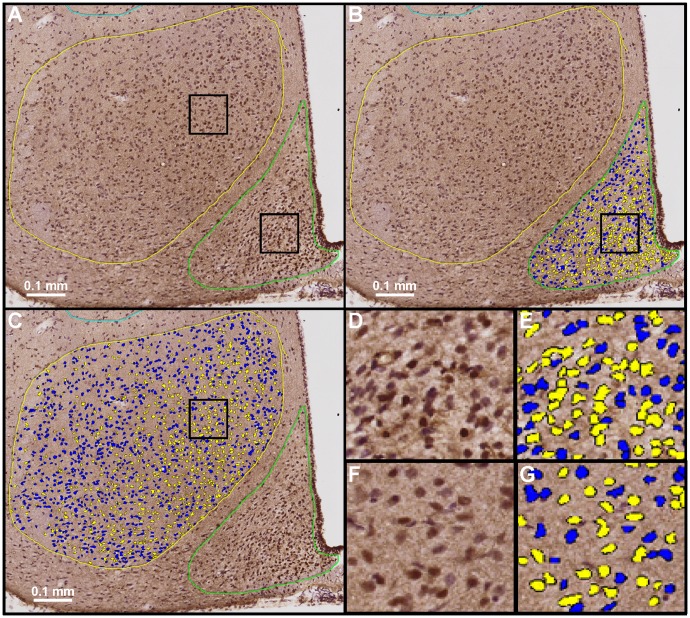
Visualization of STAT3 Phosphorylation in the Hypothalamus of LF mice. (A-C) A representative example of pSTAT3 (brown) and hematoxylin (blue) staining in the hypothalamus is presented; ARH and VMH are indicated by green and yellow outlines, respectively. pSTAT3 staining intensity analyses are indicated as an overlay for positive (yellow) and negative (blue) pSTAT3 staining for the ARH (B) and VMH (C). (D-G) Increased magnification of a 100 micron section of ARH (D-E) or VMH (F-G) are presented, with analysis overlay in panels E and G, respectively.

**Fig 4:**
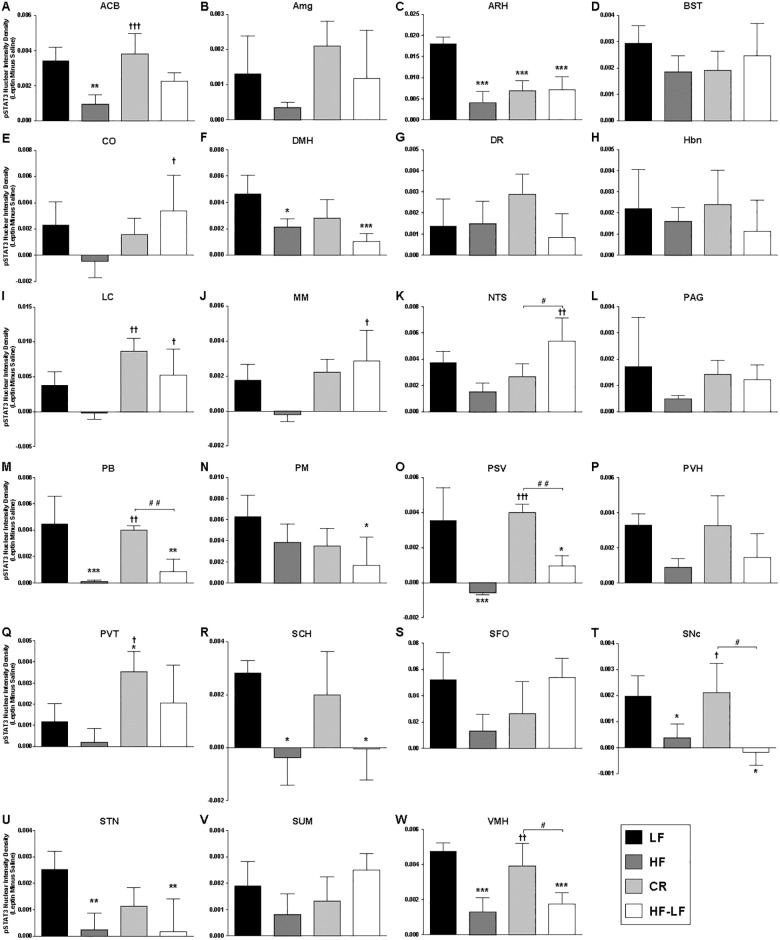
Intensity and Density of Nuclear STAT3 Phosphorylation Induced by Exogenous Leptin in Brain Regions of Weight Perturbed Mice. A summary of leptin-induced (leptin minus saline) pSTAT3 immunohistochemistry data (mean +/- SEM) is presented for all brain regions analyzed (N = 3–5 per brain region; see S2 Table); LF (black), HF (dark gray), CR (light gray), and HF-LF (white) groups are indicated in panel A. pSTAT3 Nuclear Intensity Density was calculated as described in the Methods and represents the additive signal intensity of the entire region. Brain region is identified above each graph according to S1 Table. * P<0.05, ** P<0.01, *** P<0.001 compared to LF; † P<0.05, †† P<0.01, ††† P<0.001 compared to HF; # P<0.05, ## P<0.01 between weight reduced groups (CR & HF-LF).

To assess the effects of weight perturbation on leptin receptor signaling in the brain, phosphorylation of signal transducer and activator of transcription 3 (pSTAT3) was used as a proxy for leptin receptor activation [25] and quantified in specific brain regions following a single i.p. dose of 3 mg/kg leptin (or saline) to mice fasted overnight (Fig 3; see Experimental Procedures). The relatively high dose of 3 mg/kg leptin was chosen to maximize the ability to detect and analyze regions with lower cell and/or receptor density that may have been overlooked in previous studies. Analysis of STAT3 phosphorylation revealed evidence of leptin receptor activation throughout the brain in a pattern similar to that previously reported for leptin receptor expression [26, 27]. Staining was initially quantified in 34 brain regions (selected due to presence of a moderate-to-strong pSTAT3 signal in a leptin-treated LF brain) for: LF leptin- or saline-treated; HF leptin-treated; and CR leptin-treated groups (n = 1 per treatment group; S1 Fig). Following this pilot analysis, 23 of the initial 34 brain regions were selected for further analysis by virtue of meeting one of the following criteria: 1. pSTAT3 nuclear intensity density ≥0.01 and/or 2 (see Methods). Previous implication of the brain region in the control of feeding behavior or leptin receptor signaling [26, 27]. The regions subjected to complete analysis are listed in Methods and described in S1 Table. Of these regions, the SFO and ARH displayed a much stronger pSTAT3 signal than the other regions (Fig 4 and S2 Fig).

**Fig 5:**
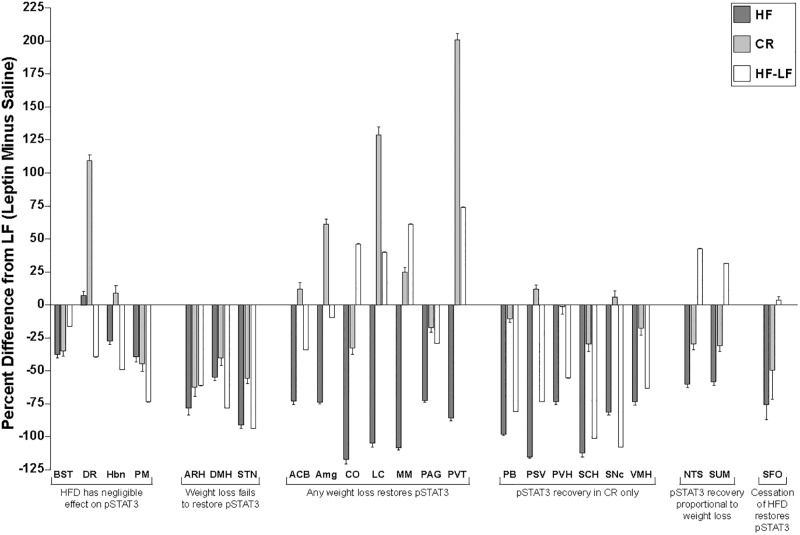
Summary of Changes in Intensity and Density of Nuclear pSTAT3 Induced by Exogenous Leptin When Compared to LF mice. Leptin-induced (leptin minus saline) pSTAT3 nuclear intensity data for weight-perturbed mice is presented; HF (dark gray), CR (light gray), and HF-LF (white) groups (as indicated in the figure legend) are presented as a percentage of LF intensity levels. † P<0.05, †† P<0.01, ††† P<0.001 compared to HF; # P<0.05, ## P<0.01 between weight reduced groups (CR & HF-LF). Brain region identity is indicated below each graph according to S1 Table.

Each of the 4 groups of mice displayed a unique pattern of STAT3 phosphorylation (Fig 4 and S2 Fig; summarized in Fig 5, Table 2 and S3 Table). When compared to LF mice, HF mice displayed >40% reduction in pSTAT3 response to exogenous leptin (as measured by the difference between the leptin-treated and saline-treated pSTAT3 values for each treatment group) in most brain regions analyzed, with only the DR (+7.1%), Hbn (-27.3%), BST (-37.5%), and PM (-38.9%) exhibiting a loss of leptin-inducible response <40%. Leptin-induced pSTAT3 levels were decreased >40% in the ACB, Amg, ARH, DMH, NTS, PAG, PVH, PVT, SFO, SNc, STN, SUM, and VMH; a >90% decrease was observed in the CO, LC, MM, PB, PSV, and SCH. (Figs 4 and 5, Table 2, and S3 Table). In some cases, this loss of leptin-inducible pSTAT3 signal in HF mice was not due to decreased visualization of colorimetric staining, but due rather to an increase in the baseline pSTAT3 signal observed in saline-treated HF animals. Six brain regions of HF animals revealed a >20% increase in basal (saline-injected) pSTAT3 staining compared to LF mice (CO, PM, SNc, ARH, PSV, and VMH), with the ARH (+62%), PSV (+83%), and VMH (90%) displaying the largest increases in basal STAT3 phosphorylation (Fig 4 and S3 Fig). Basal pSTAT3 in HF mice was also reduced >20% in the BST, Hbn, SFO, and SUM.

**Table 2:** Summary of pSTAT3 Results by Brain Region Compared to Leptin-Induced pSTAT3 Levels (Leptin minus Saline) in LF Mice.

Percent Δ vs. LF	Increased >20%	No Change*	Decreased 20–50%	Decreased 50–90%	Decreased >90%
**HF**	-----	DR	BST Hbn PM	ACB Amg ARH DMH NTS PAG PVH PVT SFO SNc SUM VMH	CO LC MM PB PSV SCH STN
**CR**	Amg DR LC PVT	ACB Hbn MM PAG PB PSV PVH SNc VMH	BST CO DMH NTS PM SCH SFO SUM	ARH STN	-----
**HF-LF**	CO LC MM NTS PVT SUM	Amg BST SFO	ACB DR Hbn PAG	ARH DMH PB PM PSV PVH VMH	SCH SNc STN

* Value was within 20% of LF leptin-induced pSTAT3 level.

Brain region abbreviations are provided in the methods and S1 Table.

In most brain regions of the CR mice, leptin-induced STAT3 phosphorylation was partially or fully restored to levels observed in brains of LF mice (Figs 4 and 5, Table 2, and S3 Table). Thus, in CR mice, pSTAT3 levels similar to those seen in LF mice were observed in the ACB, Hbn, MM, PAG, PB, PSV, PVH, SNc, and VMH, indicating that caloric restriction restores neuronal sensitivity neurons to leptin in these regions to pre-obese levels. pSTAT3 induction greater than LF was observed in the Amg (+61.3%), DR (+109.2%), LC (+128.7%), and PVT (+200.8%), suggesting that neurons in these regions were rendered hypersensitive to endogenous leptin compared to their counterparts in never-obese LF mice. pSTAT3 levels lower than LF but higher than HF were observed in the CO, SCH, SUM, and NTS, indicating a partial recovery of STAT3 phosphorylation in these regions. CR mice also displayed levels of pSTAT3 induction in the ARH, BST, DMH, PM, SFO, and STN that were similar to those observed for LF (i.e. caloric restriction did not result in recovery of leptin signaling in these six regions) (Figs 4 and 5, Table 2, and S3 Table). Elevations of basal levels of pSTAT3 in the ARH, DMH, and STN of CR mice may account in part for the lack of apparent response to exogenous leptin in these regions (Fig 4 and S3 Fig).

In mice losing weight via diet modification (HF-LF), the pSTAT3 responses to leptin in the Amg, BST, and SFO were similar to those observed in LF mice (Figs 4 and 5, Table 2, and S3 Table). Leptin-induced pSTAT3 responses of HF-LF mice were greater than LF mice in the CO, LC, MM, NTS, PVT, and SUM, suggesting that a return to a lower fat diet and/or body weight can restore or even enhance leptin signaling in these regions. In these HF-LF mice, reduced exogenous leptin sensing (compared to LF) was observed in twelve brain regions in a manner that was either independent of elevated basal STAT3 phosphorylation (DR, Hbn, PSV, PVH, SCH, and VMH) or driven, at least in part, by increased pSTAT3 levels in saline-treated controls (ARH, DMH, PB, PM, SNc, and STN) (Figs 4 and 5, Tables 1 and 2, and S3 Table). Leptin-induced pSTAT3 levels in these areas were similar to (or lower than) those observed in HF mice (regardless of changes in basal pSTAT3 levels), indicating that a combination of diet modification and weight loss are not sufficient to restore leptin-induced STAT3 phosphorylation in these twelve regions. A partial recovery of leptin-induced signaling compared with the DIO state (i.e. more leptin-induced pSTAT3 than HF but less than LF) was observed in the ACB and PAG (Figs 4 and 5, Table 2, and S3 Table).

## Discussion

We find that obesity due to chronic exposure to a high fat diet impairs sensing of leptin (as measured by STAT3 phosphorylation in response to exogenous leptin) throughout the brain, which is congruent with similar studies in rodents and humans [28–31]. Weight loss following DIO restores CNS leptin signaling to varying degrees, dependent upon brain region and the means by which weight loss is invoked (HF-LF vs. CR; Table 3 and S4 Fig). In response to acute exogenous leptin, the degree to which pSTAT3 levels increase towards to those of never-obese controls ranges from no recovery (ARH, DMH and STN for either intervention), to partial recovery (NTS and SUM for CR), to increases beyond those observed in LF mice (Amg and DR for CR group; CO, MM, NTS, and SUM for HF-LF group; LC and PVT for either weight loss group). In 12 regions leptin-induced pSTAT3 signatures failed to increase to control levels in either CR (ARH, DMH, STN, NTS, SUM and SFO) or HF-LF (ARH, DMH, STN, PB, PSV, PVH, SCH, SNc, and VMH) animals (Figs 4 and 5, Table 2, and S3 Table). Thus, a return to normal body weight/composition (regardless of diet composition) did not restore normal leptin sensing in many regions of the brain.

**Table 3:** Classification of Region-Specific Leptin-Induced pSTAT3 Responses in Weight Perturbed Versus LF Mice.

**HFD has negligible effect on pSTAT3**	**BST DR Hbn PM**
**Weight loss fails to restore pSTAT3**	**ARH DMH STN**
**Any weight loss restores pSTAT3**	**ACB Amg CO LC MM PAG PVT**
**pSTAT3 recovery in CR only**	**PB PSV PVH SCH SNc VMH**
**pSTAT3 recovery proportional to weight loss**	**NTS SUM**
**Cessation of HFD restores pSTAT3**	**SFO**

Brain region abbreviations are provided in S1 Table.

Increased constitutive STAT3 phosphorylation (in the absence of exogenous leptin administration) was also observed in multiple brain regions in weight-perturbed mice: PSV and VMH for HF; LC for CR; BST, PB, PM, PVH and SUM for HF-LF; ARH, DMH and STN for both CR and HF-LF (S2 and S3 Figs). In a recent study, endogenous leptin signaling remained intact in the ARH of DIO mice [32]. Though this finding was only reported for the ARH—and there was no leptin-treated induction of pSTAT3 for comparison to the current study—it is possible that the constitutive phosphorylation signal observed in the ARH in each study may represent a region-specific relative hypersensitivity to endogenous leptin, creating a ceiling effect beyond which exogenous leptin treatment may not further activate the STAT3 signaling pathway in specific brain regions [33]. Alternatively, these increases in STAT3 phosphorylation in the absence of exogenous leptin could be generated by a localized inflammatory cytokine response resulting from long-term HFD exposure.

Restoration of pSTAT3 responses to leptin following DIO varies by both neuroanatomical region and the manner in which weight loss is achieved. Caloric restriction of a high fat diet (CR), for example, returns leptin-induced pSTAT3 levels to those observed in LF mice [PB, PSV, PVH, SCH, SNc, VMH] or actually increases the response [Amg, LC, PVT]. Similar signal increases are observed in the LC and PVT (but not Amg) following weight loss consequent to provision of an *ad libitum* low fat diet (HF-LF). This finding is consistent with previous observations in humans that leptin responsivity—as measured by fMRI—is greater in some brain regions following weight loss than during maintenance of usual weight [12]. Hypocaloric states result in declines in leptin per unit fat mass [34, 35]. As leptin concentrations of CR mice fall on the same regression line (versus fat mass) as their *ad libitum* fed counterparts, CR mice are weight-stable but not in caloric deficit. Even though CR and HF-LF mice do not have equivalent fat mass or circulating leptin concentrations, the effects of caloric restriction compared to those of weight perturbation on STAT3 phosphorylation might be attributable solely to the protocol for caloric restriction rather than to weight loss. For instance, the twice-daily feeding regime used in the caloric restriction protocol may disrupt circadian rhythms, which could influence phenotypes specific to the CR group in regions such as the SCH, which contains the dominant systemic circadian pacemaker [36]. Alternatively, enhancement of leptin signal detection specific to the CR group could implicate these brain regions as sources of the perception of hunger and/or the relative hypometabolism observed for both CR mice [3] and weight-reduced humans [4]. As the increased palatability of the HFD can induce a hedonic response, restricting access to HFD likely affects not only leptin signaling but other aspects of feeding behavior circuitry [37]. Identification of altered leptin signaling in specific brain regions such as the SNc and ACB, sites known to control addictive and reward-based behavior [38], is consistent with previous observations of increased activity in reward circuitry following weight loss in humans [12, 39] and highlights these regions as likely sites where leptin modulates the hedonic control of food intake [40]. Additionally, the persistence of insulin resistance in CR versus HF-LF mice could influence the relative differences in leptin signaling observed for specific brain regions for these treatment groups, as leptin and insulin signaling pathways are convergent via effects on PI3 kinase [41], and increased insulin signaling has been demonstrated to inhibit leptin STAT3 signaling in the hypothalamus [42].

Diet modification (HF-LF) also restores (increases) leptin pSTAT3 responses in the NTS, SFO and SUM to levels similar to those observed in LF mice. The identification of a brain region (SFO) displaying recovery of leptin signaling in the HF-LF group (without comparable improvement following CR) identifies a brain region in which the restorative effect of weight loss on STAT3 phosphorylation in the brain is attributable primarily to diet modification. While the ability of the SFO to respond to leptin and affect energy balance has been previously described [43], its nature as a circumventricular organ, a highly vascularized region of the brain lacking a blood-brain barrier [44], may contribute to its responses to diet fat content in a unique manner when compared to the other 22 brain regions analyzed.

Though a partial recovery (increase) of leptin signaling is also observed in CR mice in the NTS and SUM, HF-LF pSTAT3 levels greatly exceed those of CR, identifying areas of the brain in which diet modification restores leptin sensing to a greater extent than caloric restriction alone. However, the presence of the partial recovery observed in these regions in the CR group (which had 20% weight loss imposed by design) suggests that the partial recovery in NTS and SUM results from weight loss rather than the diet switch, with the degree to which leptin sensing is restored being proportional to the amount of weight lost. In other words, caloric restriction of a HFD in CR mice (84.7% of calories at maximum weight) led (as designed) to limited weight loss when compared to HF-LF. Additionally, restoration of the pSTAT3 signal was proportional to the amount of weight lost, potentially identifying these regions as sites responsible for the reduced energy expenditure characterizing the CR state. A “complete” recovery of leptin sensing was observed in these regions only when weight loss brought the animal to control body weight. As weight loss in both weight-reduced groups resulted primarily from a decrease in fat mass rather than fat-free mass (Fig 2A), recovery of leptin-induced STAT3 phosphorylation in these brain regions is dependent on reductions of fat mass (and consequential reductions in circulating leptin concentrations) rather than on total weight loss.

Weight loss (regardless of method) restored STAT3 phosphorylation to levels that equaled or exceeded those of LF mice in the ACB, Amg, CO, LC, MM, PAG and PVT. The fact that weight loss by either method did not restore leptin sensing in the ARH or DMH, the hypothalamic regions containing some of the highest concentration of leptin receptor-expressing neurons in the brain [26], suggests that the ability to sense a leptin signal may be permanently reduced in these regions following long term consumption of a HFD. Though prior studies have identified HFD-induced loss of leptin signaling in the ARH but not in the DMH [16, 17], either the use of mice in the current study versus rats [16], and/or the longer period of HFD feeding (30 weeks versus 16 weeks) [17] employed in the current study could account for the reduction of leptin-induced pSTAT3 in the DMH not previously observed with HFD feeding. When combined with the fact that several brain regions show recovery of pSTAT3 responsiveness specific to caloric restriction rather than weight loss *per se*, the absence of recovery in the ARH, DMH and STN demonstrates that weight loss alone is not sufficient to restore normal leptin signal transduction throughout the brain. The differences in age and species between our current study and prior reports described above may also account for the absence of certain brain regions from our study (due to lack of consistently detectable pSTAT3 staining) in which leptin signaling has previously been demonstrated, and the inclusion of regions such as the ACB, which has been reported to have minimal leptin receptor RNA but yielded a quantifiable pSTAT3 signal.

Diet modification (HF-LF) had less impact than caloric restriction on adjusted energy expenditure (-5%, P<0.14 vs. -16%, P<0.0001, respectively), despite the fact that the degree of weight loss was much greater in the former. Similar impact of diet composition on metabolic adjustment to weight loss has been seen by us in earlier studies [3, 4]. Restricting access—even in a weight stable state—to a highly palatable diet (CR) provokes greater decline in adjusted energy expenditure than does *ad libitum* access to a less palatable (chow) diet (HF-LF). The stronger drive to eat in the CR state, and the greater reduction in adjusted energy expenditure, are likely coordinate responses mediated by the brain. The signals that inform these responses are not clear, but appreciation of region-specific differences in leptin signaling identified in this study could point to the CNS nexus for this physiology. As noted below, there is overlap between the regions affected by these manipulations in mice, and those affected by comparable manipulations in human subjects.

The reduction of previously elevated blood leptin concentrations that accompanies reduction in adiposity creates an apparent state of relative hypoleptinemia in hypothalamic (and probably other) neurons resulting in reduced energy expenditure and increasing hunger. A majority of obesity-related autonomic, behavioral, metabolic, and neuroendocrine responses in rodents and humans are mitigated or restored by leptin “replacement” [10–15]. However, decreased circulating leptin concentrations following cessation of artificially induced hyperleptinemia in the absence of obesity fails to raise the body weight “set point” in mice [19]. This finding suggests that although diet- and/or adiposity-mediated alterations in the brain may impair leptin signaling, hyperleptinemia *per se* is not primarily responsible for raising this “set point”. If not hyperleptinemia, any of the myriad metabolic consequences of obesity and/or diet composition—e.g., altered neuronal cell types [45] or connections [3], as yet unidentified adipokines [19], and hypothalamic inflammation and/or gliosis [28, 31]–could be responsible for changes in defended body weight. Additionally, the restoration of leptin sensing in specific brain regions could be either responsible for, or secondary to, the observed weight loss.

The identification of brain regions exhibiting similar effects of weight loss on pSTAT3 levels- regardless of the means of weight loss—implies some commonality in the mechanisms by which weight loss influences leptin signal transduction. However, the ability of each weight loss expedient to increase STAT3 phosphorylation in distinct brain regions demonstrates that weight loss by either caloric restriction or diet modification produces unique signatures of leptin signaling in the brain. Elucidating the molecular physiology of the leptin sensing neuron populations in each of these brain regions is essential to understanding the mechanisms by which obesity disrupts (and subsequent weight loss succeeds or fails to restore) leptin signaling in isolated brain nuclei.

It is of course possible—even likely—that a portion of the observed alterations of leptin-induced pSTAT3 in weight-perturbed groups result specifically from the introduction and manipulation of the HFD rather than weight gain or loss. Would these same responses be seen in animals weight-gained by intragastric infusion of a diet comparable in fat content to that of standard mouse chow? Would 20% weight reduction of a HFD mouse using feeding of standard chow followed by weight-stabilization using gastric infusion result in the same changes seen in the CR animals reported here? Such “controls” are technically difficult and would introduce other confounds, but these concerns are relevant to the interpretation of these studies. Finally, for regions in which leptin-induced STAT3 phosphorylation levels do not fluctuate in proportion to weight gain or loss, secretion of molecules other than leptin from adipose or other tissues could modulate the ability of individual brain regions to respond to leptin [46].

## Conclusions

In summary, a high fat diet attenuates leptin signaling throughout the brain in a region-specific manner, but some brain regions maintain their ability to sense leptin despite the deleterious effects of HFD. Weight loss restores leptin sensitivity to some degree in most brain regions (but not all), while other brain regions display hypersensitivity to leptin following weight loss. In several brain regions, normal leptin responsivity was restored by weight loss, with the pattern of restoration dependent on the method of weight loss. Therefore, the effects of diet on leptin signaling in the brain vary by brain region, with the direction and magnitude by which diet affects leptin signaling dependent on the method of weight loss.

## Supporting Information

S1 Fig pSTAT3 Nuclear Intensity Density. A summary of pSTAT3 immunohistochemistry nuclear intensity density is presented for all brain regions analyzed in the pilot study. LF-Saline, LF-Leptin, HF-Leptin, and CR-Leptin treated mice are indicated by diagonal, black, dark gray, and light gray bars, respectively. Brain region identity is indicated above each graph according to S1 Table.
(PDF)

S2 Fig pSTAT3 Nuclear Intensity Density Raw Values. A summary of pSTAT3 immunohistochemistry nuclear intensity density is presented for all brain regions analyzed. LF, HF, CR and HF-LF groups are indicated by black, dark gray, light gray and white bars, respectively; saline- and leptin-treated values are indicated by diagonal lines and solid bars, respectively. Brain region identity is indicated above each graph according to Table S1. * P<0.05, ** P<0.01 compared to CON-AL; † P<0.05, †† P<0.01 compared to DIO-AL; # P<0.05, ## P<0.01 between weight reduced groups; § P<0.05, §§ P<0.01 between saline- and leptin-treated mice within a treatment group.
(PDF)

S3 Fig Summary of Changes in Intensity and Density of Basal Nuclear pSTAT3 When Compared to LF mice. Leptin-induced (leptin minus saline) pSTAT3 nuclear intensity data for weight-perturbed mice is presented; HF (dark gray), CR (light gray), and HF-LF (white) groups (as indicated in the figure legend) are presented as a percentage of LF intensity levels. * P<0.05 compared to LF; # P<0.05 between weight reduced groups (CR & HF-LF). Brain region identity is indicated below each graph according to S1 Table.
(PDF)

S4 Fig Summary of Region-Specific Leptin-Induced pSTAT3 Response to Weight Perturbations in Mice. A Venn diagram is presented summarizing the results from Fig 5. Brain regions in which leptin-induced pSTAT3 was increased >50% above LF levels following weight loss are indicated in white text.
(PDF)

S1 Table Brain Region Abbreviations. (PDF)

S2 Table Number of mice included in PSTAT3 analysis for each mouse group and brain region. (PDF)

S3 Table Summary of Changes in Intensity and Density of Nuclear pSTAT3 Induced by Exogenous Leptin. (PDF)
