# Supplementary material for: Weight Perturbation Alters Leptin Signal Transduction in a Region-Specific Manner throughout the Brain
Source: PLoS One. 2017 Jan 20;12(1):e0168226. doi: 10.1371/journal.pone.0168226 (PMC5249166; doi:10.1371/journal.pone.0168226)
Supplement: S1 Fig — A summary of pSTAT3 immunohistochemistry nuclear intensity density is presented for all brain regions analyzed in the pilot study. LF-Saline, LF-Leptin, HF-Leptin, and CR-Leptin treated mice are indicated by diagonal, black, dark gray, and light gray bars, respectively. Brain region identity is indicated above each graph according to S1 Table. (PDF) [file pone.0168226.s001.pdf]

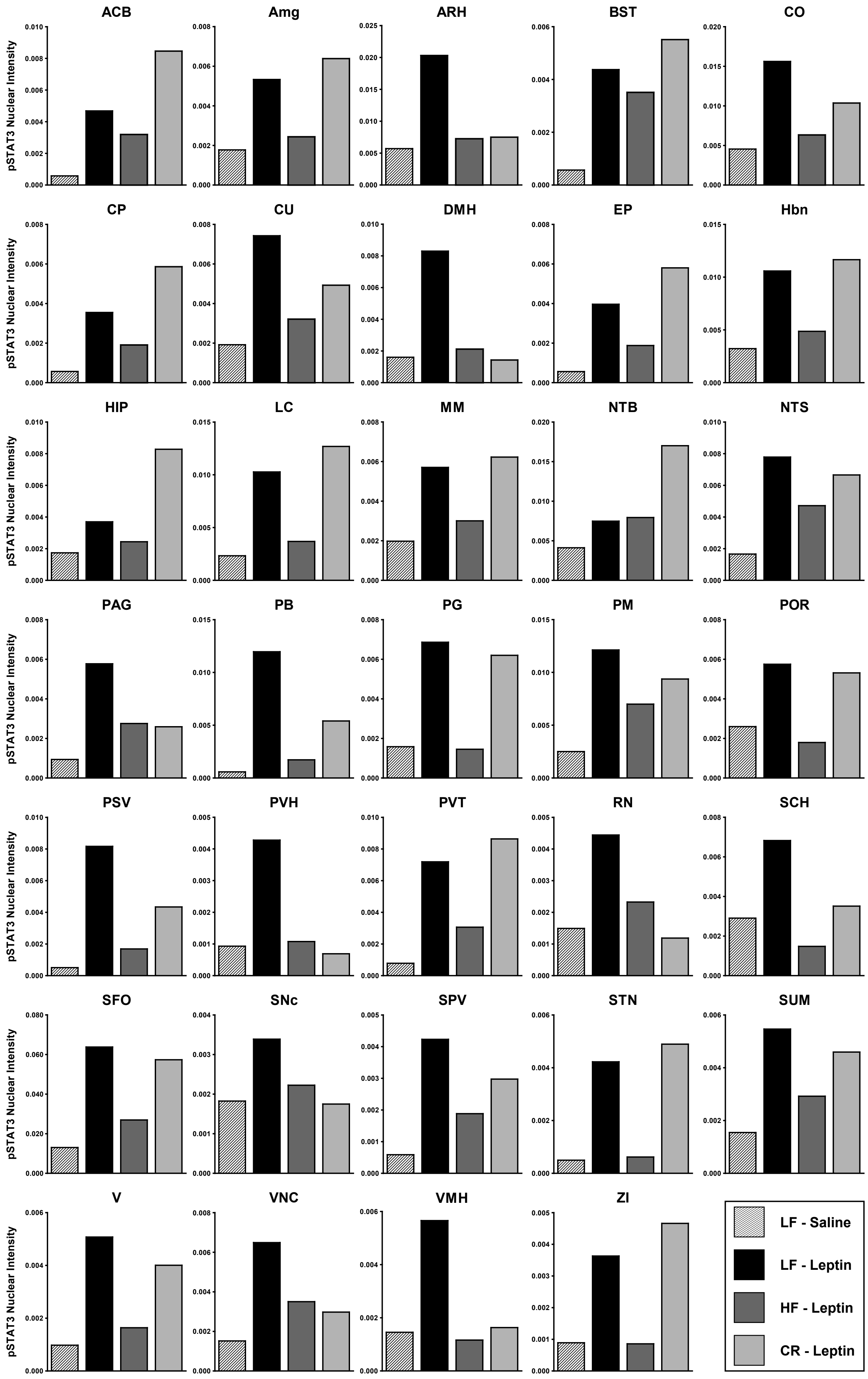

S1 Fig – pSTAT3 Nuclear Intensity Density. A summary of pSTAT3 immunohistochemistry nuclear intensity density is presented for all brain regions analyzed in the pilot study. LF-Saline, LF-Leptin, HF-Leptin, and CR-Leptin treated mice are indicated by diagonal, black, dark gray, and light gray bars, respectively. Brain region identity is indicated above each graph according to S1 Table.

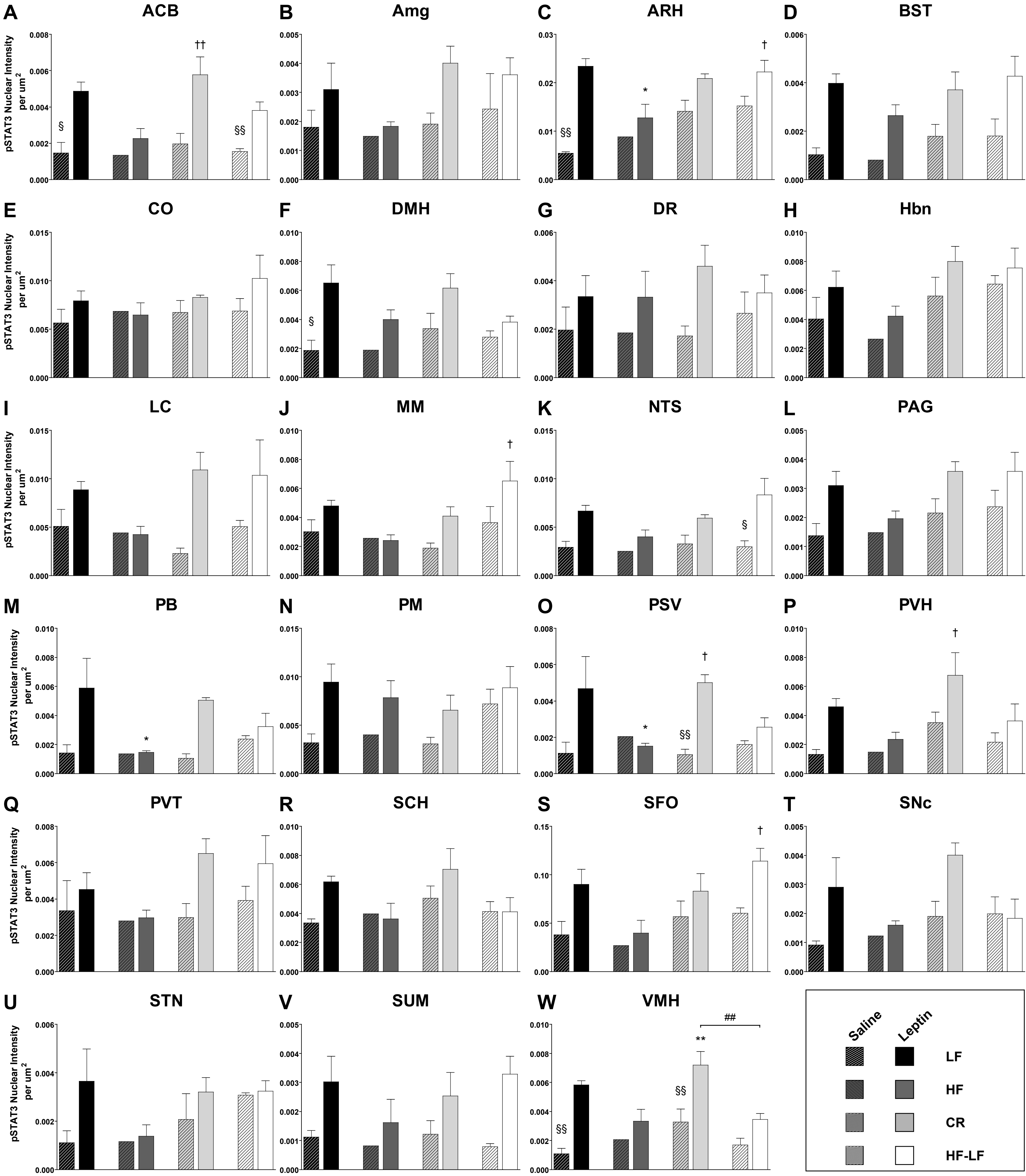

S2 Fig – pSTAT3 Nuclear Intensity Density Raw Values. A summary of pSTAT3 immunohistochemistry nuclear intensity density is presented for all brain regions analyzed. LF, HF, CR and HF-LF groups are indicated by black, dark gray, light gray and white bars, respectively; saline- and leptin-treated values are indicated by diagonal lines and solid bars, respectively. Brain region identity is indicated above each graph according to Table S1. * P<0.05, ** P<0.01 compared to CON-AL; † P<0.05, †† P<0.01 compared to DIO-AL; # P<0.05, ## P<0.01 between weight reduced groups; § P<0.05, §§ P<0.01 between saline- and leptin-treated mice within a treatment group.

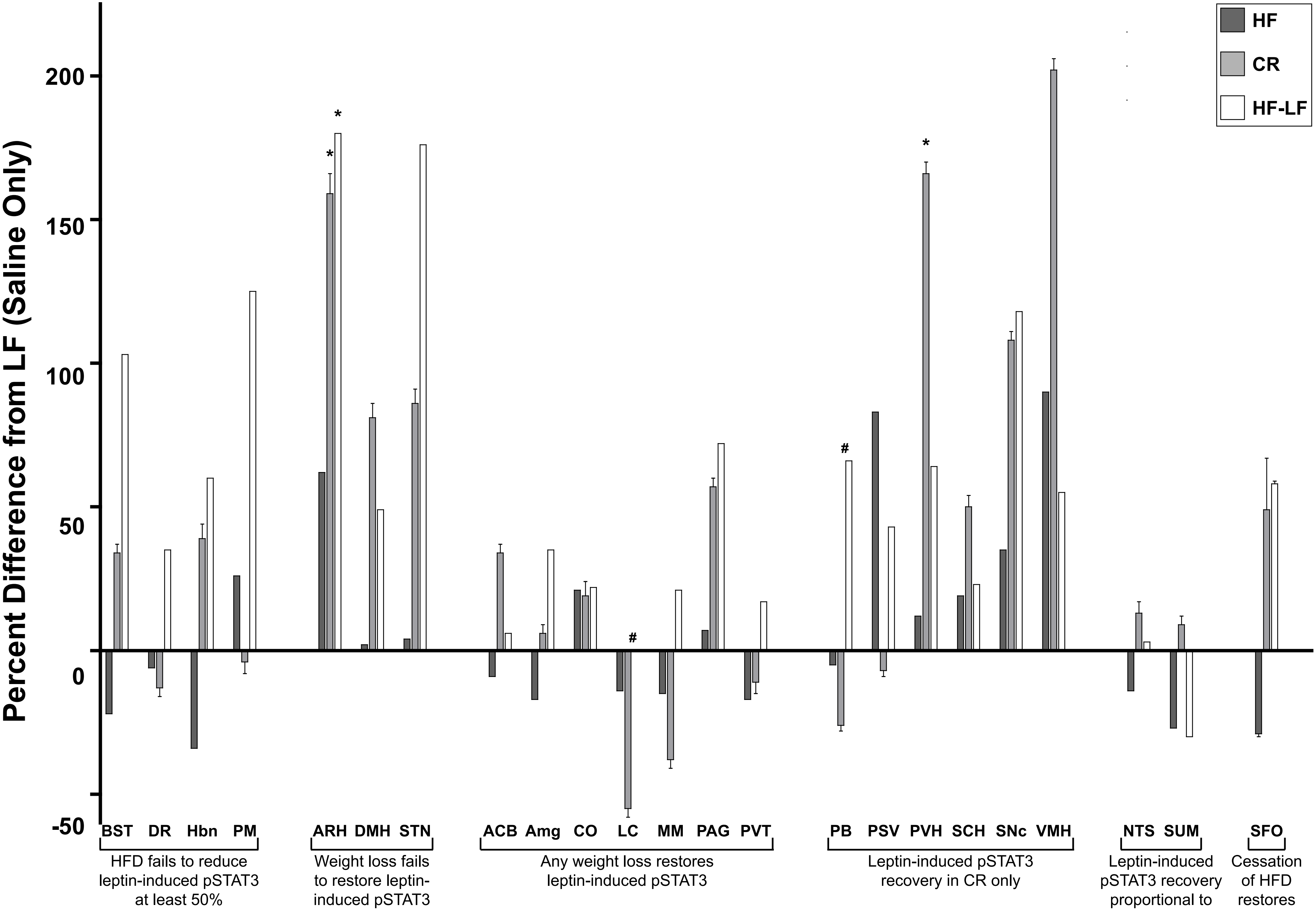

S3 Fig – Summary of Changes in Intensity and Density of Basal Nuclear pSTAT3 When Compared to LF mice. Leptin-induced (leptin minus saline) pSTAT3 nuclear intensity data for weight-perturbed mice is presented; HF (dark gray), CR (light gray), and HF-LF (white) groups (as indicated in the figure legend) are presented as a percentage of LF intensity levels. * P<0.05 compared to LF; # P<0.05 between weight reduced groups (CR & HF-LF). Brain region identity is indicated below each graph according to S1 Table.

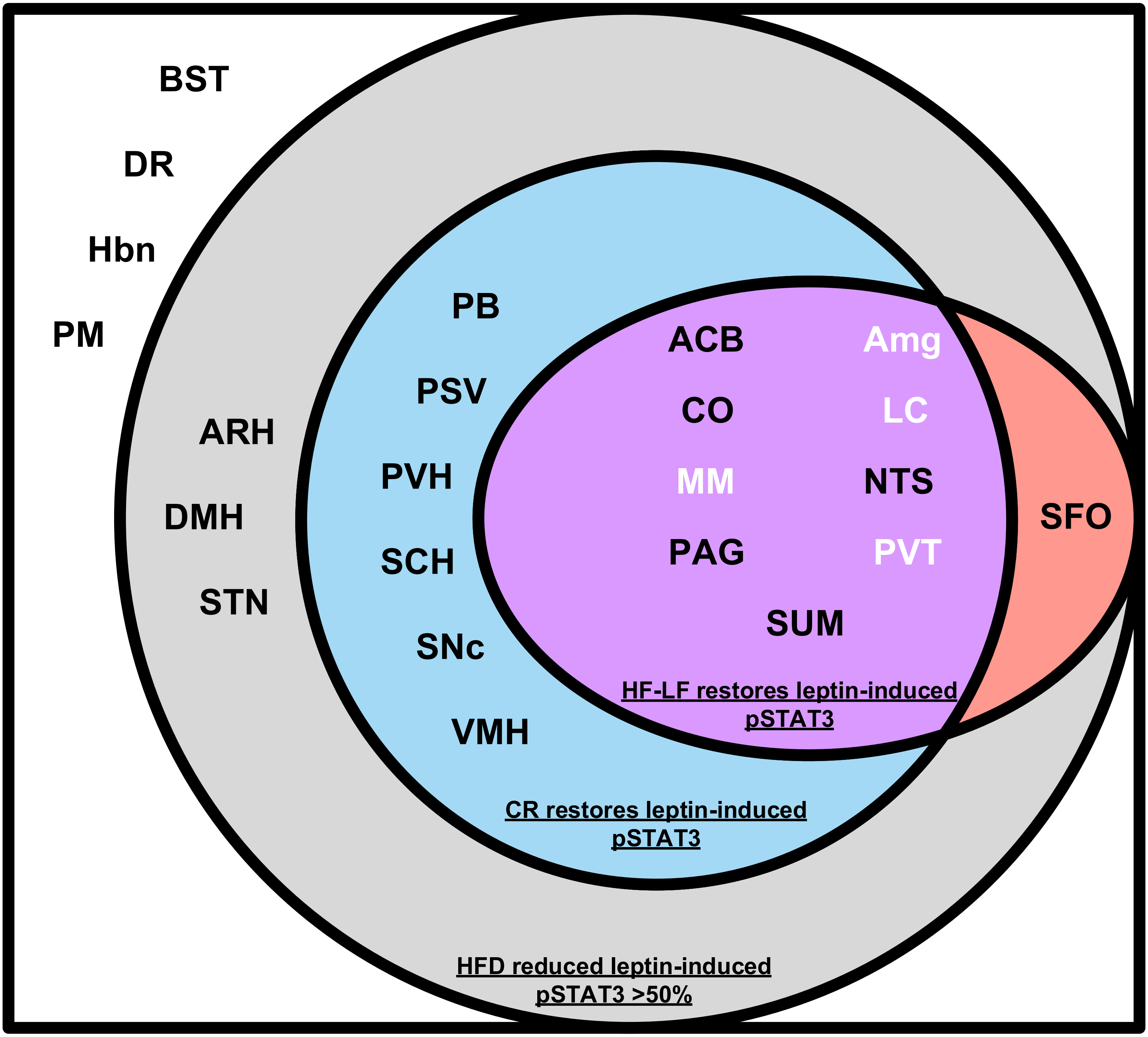

S4 Fig – Summary of Region-Specific Leptin-Induced pSTAT3 Response to Weight Perturbations in Mice. A Venn diagram is presented summarizing the results from Fig 5. Brain regions in which leptin-induced pSTAT3 was increased >50% above LF levels following weight loss are indicated in white text.

S1 Table – Brain Region Abbreviations

Brain Structure	Abbreviation	Region Name
**Cerebral Cortex**	EP	Endopiriform Nucleus
**Limbic System**	Amg	Amygdaloid Nucleus (Amygdala)*
	HIP	Hippocampus
**Striatum**	ACB	Nucleus Accumbens*
	CP	Caudoputamen
**Pallidum**	BST	Bed Nucleus of the Stria Terminalis*
**Thalamus**	Hbn	Habenula*
	PVT	Paraventricular Nucleus*
**Hypothalamus**	ARH	Arcuate Hypothalamic Nucleus*
	DMH	Dorsomedial Nucleus of the Hypothalamus*
	MM	Medial Mammillary Nucleus*
	PM	Premammillary Nucleus*
	PVH	Paraventricular Hypothalamic Nucleus*
	SCH	Suprachiasmatic Nucleus*
	STN	Subthalamic Nucleus*
	SUM	Supramammillary Nucleus*
	VMH	Ventromedial Hypothalamic Nucleus*
	ZI	Zona Incerta
**Midbrain**	DR	Dorsal Raphe Nucleus*
	PAG	Periaqueductal Gray*
	RN	Red Nucleus
	SNc	Substantia Nigra, pars compacta*
**Pons**	LC	Locus Ceruleus*
	PB	Parabrachial Nucleus*
	PG	Pontine Gray
	POR	Periolivary Region
	PSV	Principal Sensory Nucleus of the Trigeminal*
	V	Motor Nucleus of the Trigeminal
**Medulla**	CO	Cochlear Nucleus*
	CU	Cuneate Nucleus
	NTB	Nucleus of the Trapezoid Body
	NTS	Nucleus of the Solitary Tract*
	SPV	Spinal Nuclei of the Trigeminal
	VNC	Vestibular Nuclei
**Circumventricular Organs**	SFO	Subfornical Organ*

* - Denotes region selected for detailed analysis

S2 Table – Number of mice included in PSTAT3 analysis for each mouse group and brain region.

	LF	HF	CR	HF-LF
**ACB**	4	4	4	5
**Amg**	5	4	4	5
**ARH**	4	4	4	5
**BST**	4	4	4	5
**CO**	5	4	4	5
**DMH**	5	4	4	5
**DR**	5	4	4	5
**Hbn**	5	4	4	5
**LC**	4	4	4	5
**MM**	4	3	5	4
**NTS**	4	4	4	5
**PAG**	5	4	4	5
**PB**	4	4	4	5
**PM**	5	4	4	5
**PSV**	3	4	3	5
**PVH**	4	4	4	5
**PVT**	5	4	4	5
**SCH**	4	4	4	5
**SFO**	4	4	4	4
**SNc**	5	4	4	3
**STN**	5	4	4	5
**SUM**	4	3	5	4
**VMH**	4	4	4	5

S3 Table – Summary of Changes in Intensity and Density of Nuclear pSTAT3 Induced by Exogenous Leptin.

Effect of Diet/Weight Manipulation	Brain Region	HF	CR	HF-LF
HFD has negligible effect on pSTAT3	BST	-37.5% ±2.5	-34.7% ±3.8	-16.0% ±0.1
	DR	7.1% ±3.3	109.2% ±4.4	-39.1% ±0.2
	Hbn	-27.3% ±2.6	8.9% ±5.8	-48.7% ±0.2
	PM	-38.9% ±4.2	-44.4% ±5.8	-73.3% ±0.3
Weight loss fails to restore pSTAT3	ARH	-78.0% ±5.3	-62.1% ±7	-60.6% ±0.4
	DMH	-54.7% ±2.6	-40.1% ±5.4	-77.8% ±0.2
	STN	-90.9% ±2.5	-55.4% ±3.8	-93.4% ±0.1
Any weight loss restores pSTAT3	ACB	-72.9% ±2.3	11.9% ±4.8	-33.8% ±0.1
	Amg	-73.7% ±1.3	61.3% ±3.7	-9.3% ±0.2
	CO	-116.9% ±3.6	-32.6% ±5	46.2% ±0.3
	LC	-104.7% ±2.9	128.7% ±6.2	39.9% ±0.4
	MM	-108.0% ±2	24.7% ±3.8	61.3% ±0.2
	PAG	-72.1% ±1.6	-17.1% ±3.4	-28.8% ±0.1
	PVT	-85.4% ±2.1	200.8% ±4.7	73.9% ±0.3
pSTAT3 recovery in CR only	PB	-97.7% ±1.1	-10.5% ±2.7	-80.5% ±0.2
	PSV	-114.9% ±1.2	11.8% ±3.2	-73.0% ±0.2
	PVH	-73.0% ±2.2	-0.8% ±5.9	-55.1% ±0.1
	SCH	-112.1% ±3.2	-29.2% ±5.7	-100.8% ±0.1
	SNc	-81.2% ±2.3	5.8% ±4.8	-107.7% ±0.1
	VMH	-73.0% ±2.9	-17.5% ±5.1	-63.1% ±0.1
pSTAT3 recovery proportional to weight loss	NTS	-59.8% ±2.6	-29.2% ±4.4	42.6% ±0.2
	SUM	-57.9% ±2.8	-30.7% ±4.3	31.4% ±0.1
Cessation of HFD restores pSTAT3	SFO	-75.2% ±11.7	-49.1% ±22.1	3.6% ±2.5
