# Supplementary material for: Weight Perturbation Alters Leptin Signal Transduction in a Region-Specific Manner throughout the Brain
Source: PLoS One. 2017 Jan 20;12(1):e0168226. doi: 10.1371/journal.pone.0168226 (PMC5249166; doi:10.1371/journal.pone.0168226)
Supplement: S2 Fig — A summary of pSTAT3 immunohistochemistry nuclear intensity density is presented for all brain regions analyzed. LF, HF, CR and HF-LF groups are indicated by black, dark gray, light gray and white bars, respectively; saline- and leptin-treated values are indicated by diagonal lines and solid bars, respectively. Brain region identity is indicated above each graph according to Table S1. * P<0.05, ** P<0.01 compared to CON-AL; † P<0.05, †† P<0.01 compared to DIO-AL; # P<0.05, ## P<0.01 between weight reduced groups; § P<0.05, §§ P<0.01 between saline- and leptin-treated mice within a treatment group. (PDF) [file pone.0168226.s002.pdf]

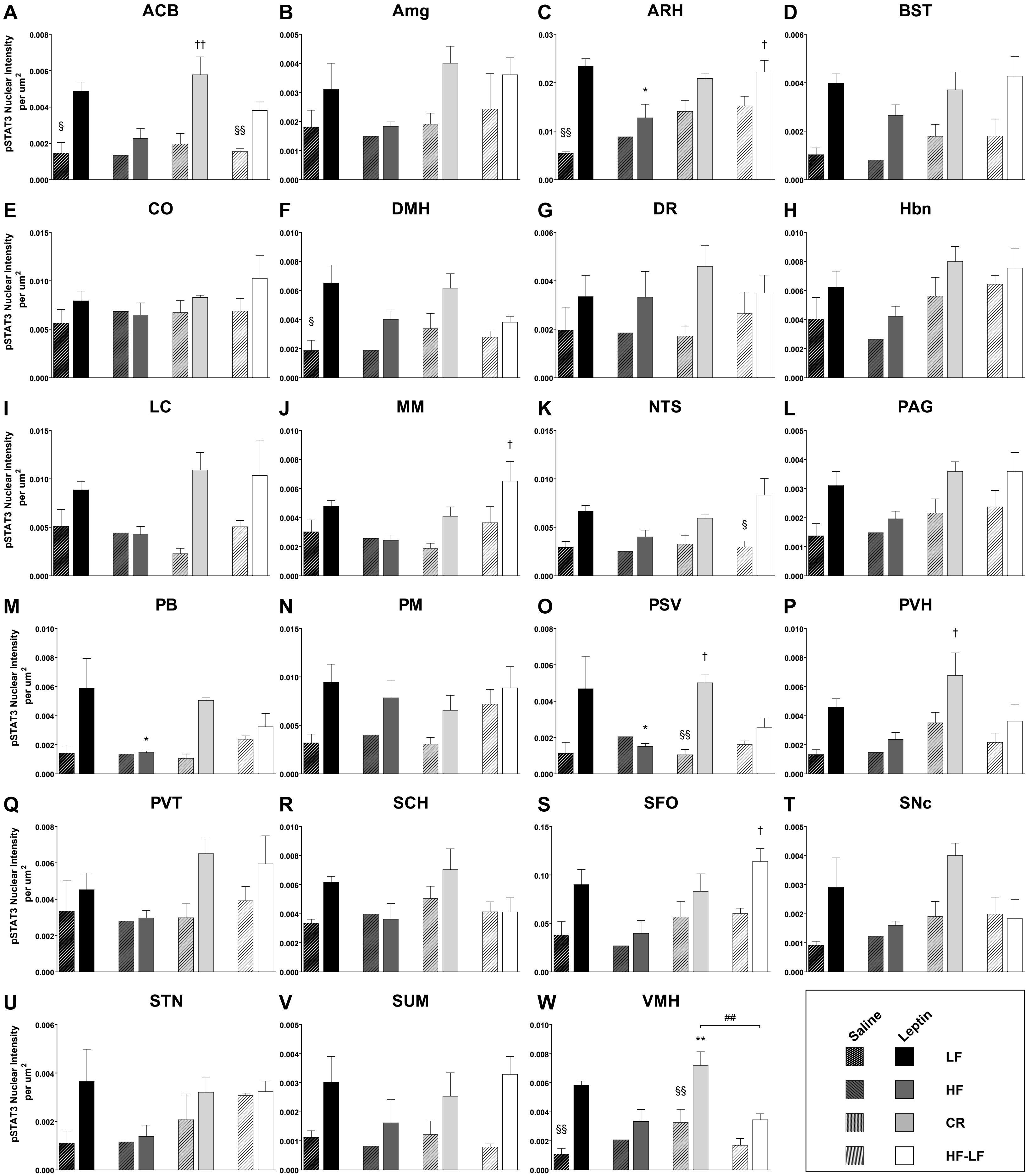

S2 Fig – pSTAT3 Nuclear Intensity Density Raw Values. A summary of pSTAT3 immunohistochemistry nuclear intensity density is presented for all brain regions analyzed. LF, HF, CR and HF-LF groups are indicated by black, dark gray, light gray and white bars, respectively; saline- and leptin-treated values are indicated by diagonal lines and solid bars, respectively. Brain region identity is indicated above each graph according to Table S1. * P<0.05, ** P<0.01 compared to CON-AL; † P<0.05, †† P<0.01 compared to DIO-AL; # P<0.05, ## P<0.01 between weight reduced groups; § P<0.05, §§ P<0.01 between saline- and leptin-treated mice within a treatment group.
