# Supplementary material for: Weight Perturbation Alters Leptin Signal Transduction in a Region-Specific Manner throughout the Brain
Source: PLoS One. 2017 Jan 20;12(1):e0168226. doi: 10.1371/journal.pone.0168226 (PMC5249166; doi:10.1371/journal.pone.0168226)
Supplement: S3 Fig — Leptin-induced (leptin minus saline) pSTAT3 nuclear intensity data for weight-perturbed mice is presented; HF (dark gray), CR (light gray), and HF-LF (white) groups (as indicated in the figure legend) are presented as a percentage of LF intensity levels. * P<0.05 compared to LF; # P<0.05 between weight reduced groups (CR & HF-LF). Brain region identity is indicated below each graph according to S1 Table. (PDF) [file pone.0168226.s003.pdf]

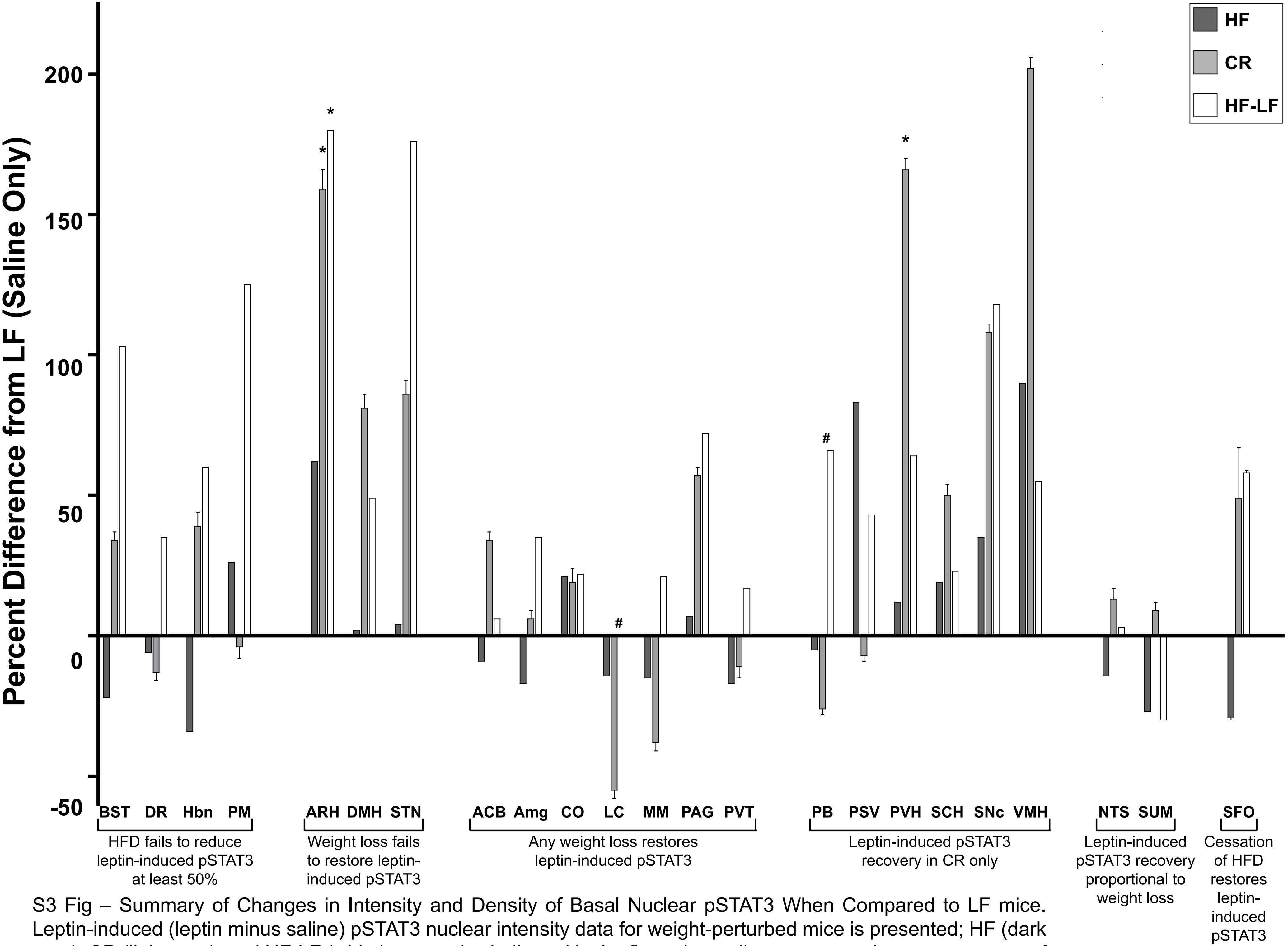

S3 Fig – Summary of Changes in Intensity and Density of Basal Nuclear pSTAT3 When Compared to LF mice. Leptin-induced (leptin minus saline) pSTAT3 nuclear intensity data for weight-perturbed mice is presented; HF (dark gray), CR (light gray), and HF-LF (white) groups (as indicated in the figure legend) are presented as a percentage of LF intensity levels. * $P<0.05$ compared to LF; # $P<0.05$ between weight reduced groups (CR & HF-LF). Brain region identity is indicated below each graph according to S1 Table.
