# Supplementary material for: Weight Perturbation Alters Leptin Signal Transduction in a Region-Specific Manner throughout the Brain
Source: PLoS One. 2017 Jan 20;12(1):e0168226. doi: 10.1371/journal.pone.0168226 (PMC5249166; doi:10.1371/journal.pone.0168226)
Supplement: S4 Fig — A Venn diagram is presented summarizing the results from Fig 5. Brain regions in which leptin-induced pSTAT3 was increased >50% above LF levels following weight loss are indicated in white text. (PDF) [file pone.0168226.s004.pdf]

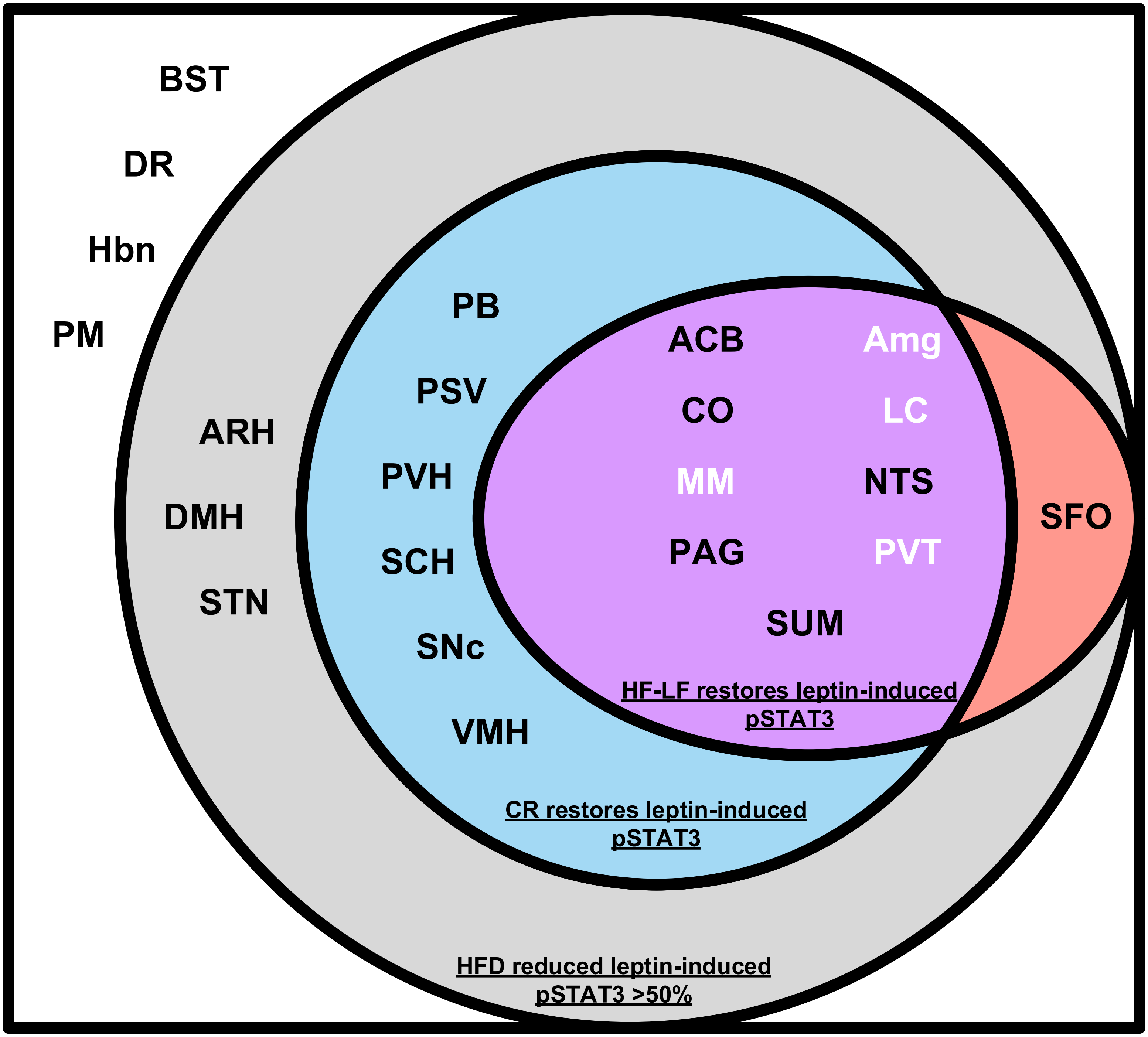

S4 Fig – Summary of Region-Specific Leptin-Induced pSTAT3 Response to Weight Perturbations in Mice. A Venn diagram is presented summarizing the results from Fig 5. Brain regions in which leptin-induced pSTAT3 was increased >50% above LF levels following weight loss are indicated in white text.
