# Supplementary material for: Weight Perturbation Alters Leptin Signal Transduction in a Region-Specific Manner throughout the Brain
Source: PLoS One. 2017 Jan 20;12(1):e0168226. doi: 10.1371/journal.pone.0168226 (PMC5249166; doi:10.1371/journal.pone.0168226)
Supplement: S1 Table — (PDF) [file pone.0168226.s005.pdf]

S1 Table – Brain Region Abbreviations

Brain Structure	Abbreviation	Region Name
**Cerebral Cortex**	EP	Endopiriform Nucleus
**Limbic System**	Amg	Amygdaloid Nucleus (Amygdala)*
	HIP	Hippocampus
**Striatum**	ACB	Nucleus Accumbens*
	CP	Caudoputamen
**Pallidum**	BST	Bed Nucleus of the Stria Terminalis*
**Thalamus**	Hbn	Habenula*
	PVT	Paraventricular Nucleus*
**Hypothalamus**	ARH	Arcuate Hypothalamic Nucleus*
	DMH	Dorsomedial Nucleus of the Hypothalamus*
	MM	Medial Mammillary Nucleus*
	PM	Premammillary Nucleus*
	PVH	Paraventricular Hypothalamic Nucleus*
	SCH	Suprachiasmatic Nucleus*
	STN	Subthalamic Nucleus*
	SUM	Supramammillary Nucleus*
	VMH	Ventromedial Hypothalamic Nucleus*
	ZI	Zona Incerta
**Midbrain**	DR	Dorsal Raphe Nucleus*
	PAG	Periaqueductal Gray*
	RN	Red Nucleus
	SNc	Substantia Nigra, pars compacta*
**Pons**	LC	Locus Ceruleus*
	PB	Parabrachial Nucleus*
	PG	Pontine Gray
	POR	Periolivary Region
	PSV	Principal Sensory Nucleus of the Trigeminal*
	V	Motor Nucleus of the Trigeminal
**Medulla**	CO	Cochlear Nucleus*
	CU	Cuneate Nucleus
	NTB	Nucleus of the Trapezoid Body
	NTS	Nucleus of the Solitary Tract*
	SPV	Spinal Nuclei of the Trigeminal
	VNC	Vestibular Nuclei
**Circumventricular Organs**	SFO	Subfornical Organ*

* - Denotes region selected for detailed analysis
