# Supplementary material for: Weight Perturbation Alters Leptin Signal Transduction in a Region-Specific Manner throughout the Brain
Source: PLoS One. 2017 Jan 20;12(1):e0168226. doi: 10.1371/journal.pone.0168226 (PMC5249166; doi:10.1371/journal.pone.0168226)
Supplement: S2 Table — (PDF) [file pone.0168226.s006.pdf]

S2 Table – Number of mice included in PSTAT3 analysis for each mouse group and brain region.

	LF	HF	CR	HF-LF
**ACB**	4	4	4	5
**Amg**	5	4	4	5
**ARH**	4	4	4	5
**BST**	4	4	4	5
**CO**	5	4	4	5
**DMH**	5	4	4	5
**DR**	5	4	4	5
**Hbn**	5	4	4	5
**LC**	4	4	4	5
**MM**	4	3	5	4
**NTS**	4	4	4	5
**PAG**	5	4	4	5
**PB**	4	4	4	5
**PM**	5	4	4	5
**PSV**	3	4	3	5
**PVH**	4	4	4	5
**PVT**	5	4	4	5
**SCH**	4	4	4	5
**SFO**	4	4	4	4
**SNc**	5	4	4	3
**STN**	5	4	4	5
**SUM**	4	3	5	4
**VMH**	4	4	4	5
