# Supplementary material for: Weight Perturbation Alters Leptin Signal Transduction in a Region-Specific Manner throughout the Brain
Source: PLoS One. 2017 Jan 20;12(1):e0168226. doi: 10.1371/journal.pone.0168226 (PMC5249166; doi:10.1371/journal.pone.0168226)
Supplement: S3 Table — (PDF) [file pone.0168226.s007.pdf]

S3 Table – Summary of Changes in Intensity and Density of Nuclear pSTAT3 Induced by Exogenous Leptin.

Effect of Diet/Weight Manipulation	Brain Region	HF	CR	HF-LF
HFD has negligible effect on pSTAT3	BST	-37.5% ±2.5	-34.7% ±3.8	-16.0% ±0.1
	DR	7.1% ±3.3	109.2% ±4.4	-39.1% ±0.2
	Hbn	-27.3% ±2.6	8.9% ±5.8	-48.7% ±0.2
	PM	-38.9% ±4.2	-44.4% ±5.8	-73.3% ±0.3
Weight loss fails to restore pSTAT3	ARH	-78.0% ±5.3	-62.1% ±7	-60.6% ±0.4
	DMH	-54.7% ±2.6	-40.1% ±5.4	-77.8% ±0.2
	STN	-90.9% ±2.5	-55.4% ±3.8	-93.4% ±0.1
Any weight loss restores pSTAT3	ACB	-72.9% ±2.3	11.9% ±4.8	-33.8% ±0.1
	Amg	-73.7% ±1.3	61.3% ±3.7	-9.3% ±0.2
	CO	-116.9% ±3.6	-32.6% ±5	46.2% ±0.3
	LC	-104.7% ±2.9	128.7% ±6.2	39.9% ±0.4
	MM	-108.0% ±2	24.7% ±3.8	61.3% ±0.2
	PAG	-72.1% ±1.6	-17.1% ±3.4	-28.8% ±0.1
	PVT	-85.4% ±2.1	200.8% ±4.7	73.9% ±0.3
pSTAT3 recovery in CR only	PB	-97.7% ±1.1	-10.5% ±2.7	-80.5% ±0.2
	PSV	-114.9% ±1.2	11.8% ±3.2	-73.0% ±0.2
	PVH	-73.0% ±2.2	-0.8% ±5.9	-55.1% ±0.1
	SCH	-112.1% ±3.2	-29.2% ±5.7	-100.8% ±0.1
	SNc	-81.2% ±2.3	5.8% ±4.8	-107.7% ±0.1
	VMH	-73.0% ±2.9	-17.5% ±5.1	-63.1% ±0.1
pSTAT3 recovery proportional to weight loss	NTS	-59.8% ±2.6	-29.2% ±4.4	42.6% ±0.2
	SUM	-57.9% ±2.8	-30.7% ±4.3	31.4% ±0.1
Cessation of HFD restores pSTAT3	SFO	-75.2% ±11.7	-49.1% ±22.1	3.6% ±2.5
